# Effects and costs of implementing predictive risk stratification in primary care: a randomised stepped wedge trial

**DOI:** 10.1136/bmjqs-2018-007976

**Published:** 2018-11-05

**Authors:** Helen Snooks, Kerry Bailey-Jones, Deborah Burge-Jones, Jeremy Dale, Jan Davies, Bridie Angela Evans, Angela Farr, Deborah Fitzsimmons, Martin Heaven, Helen Howson, Hayley Hutchings, Gareth John, Mark Kingston, Leo Lewis, Ceri Phillips, Alison Porter, Bernadette Sewell, Daniel Warm, Alan Watkins, Shirley Whitman, Victoria Williams, Ian Russell

**Affiliations:** 1 Medical School, Swansea University, Swansea, UK; 2 Abertawe Bro Morgannwg University Health Board, Neath Port Talbot, UK; 3 Warwick Medical School, University of Warwick, Coventry, UK; 4 Independent service user, Cardiff, UK; 5 Swansea Centre for Health Economics, Swansea University, Swansea, UK; 6 Bevan Commission, Swansea, UK; 7 NHS Wales Informatics Service, Cardiff, UK; 8 International Foundation for Integrated Care, Oxford, UK; 9 Hywel Dda University Health Board, Carmarthen, UK

**Keywords:** cluster trials, emergency department, health services research, primary care, cost-effectiveness

## Abstract

**Aim:**

We evaluated the introduction of a predictive risk stratification model (PRISM) into primary care. Contemporaneously National Health Service (NHS) Wales introduced Quality and Outcomes Framework payments to general practices to focus care on those at highest risk of emergency admission to hospital. The aim of this study was to evaluate the costs and effects of introducing PRISM into primary care.

**Methods:**

Randomised stepped wedge trial with 32 general practices in one Welsh health board. The intervention comprised: PRISM software; practice-based training; clinical support through two ‘general practitioner (GP) champions’ and technical support. The primary outcome was emergency hospital admissions.

**Results:**

Across 230 099 participants, PRISM implementation increased use of health services: emergency hospital admission rates by 1 % when untransformed (while change in log-transformed rate Δ_L_=0.011, 95% CI 0.010 to 0.013); emergency department (ED) attendance rates by untransformed 3 % (while Δ_L_=0.030, 95% CI 0.028 to 0.032); outpatient visit rates by untransformed 5 % (while Δ_L_=0.055, 95% CI 0.051 to 0.058); the proportion of days with recorded GP activity by untransformed 1 % (while Δ_L_=0.011, 95% CI 0.007 to 0.014) and time in hospital by untransformed 3 % (while Δ_L_=0.029, 95% CI 0.026 to 0.031). Thus NHS costs per participant increased by £76 (95% CI £46 to £106).

**Conclusions:**

Introduction of PRISM resulted in a statistically significant increase in emergency hospital admissions and use of other NHS services without evidence of benefits to patients or the NHS.

## Introduction

The ageing population with rising prevalence of chronic conditions makes unprecedented demands on healthcare services.^1 2^ In 2012–2013, there were 5.3 million emergency admissions to hospitals in England costing approximately £12.5 billion.^3^ Around half of these admissions arise from 5% of the population—typically older people with comorbidities.^4^ Patients with chronic conditions are more likely to experience emergency hospital admissions for potentially avoidable causes.^5^ An emergency admission to hospital is disruptive and unsettling, exposing patients to clinical and psychological risks and increasing their dependency.^6^

An estimated one in five emergency admissions is avoidable,^7^ especially when they arise from conditions amenable to community prevention or care.^8^ Across Europe, policies have recommended that health providers use predictive risk stratification modelling to identify patients at high risk of emergency admission to hospital for proactive management.^9–12^ In estimating individual risk scores, models typically include predictors relating to past use of healthcare, diagnoses and medications. The targeting of services at people at the highest levels of risk has been prominent in UK government policy over the past decade, notably within integrated care initiatives.^13 14^ The National Health Service (NHS) England enhanced service, “Avoiding unplanned admissions: proactive case finding and patient review for vulnerable people”, committed £480 million over 2014–2017^15^ for general practices to create registers of patients at high risk of unplanned admissions for proactive case management. Over 95% of practices participated, most using predictive risk tools to identify patients for case management. Unfortunately, a planned national evaluation of this enhanced service did not happen.^15^

In Wales, the Quality and Outcomes Framework (QOF) provided similar funding for practices to identify 0.5% of their patients at significant risk of emergency admission for clinical review and active management. Practices had to nominate a lead clinician and appropriate review dates for identified patients.^16^ However, the evidence base for the impact of such initiatives on the quality and safety of care for this patient group is weak. There is no systematic review of interventions using emergency admission risk prediction models. Though there is a review of the effectiveness of case management for high-risk patients, most included studies did not use risk models to identify patients.^17^

Hence, we aimed to evaluate the costs and effects of introducing an emergency admission risk prediction tool (PRISM) within primary care in urban South Wales.

## Methods

### Study design, participants, randomisation and masking

**Figure 1:**
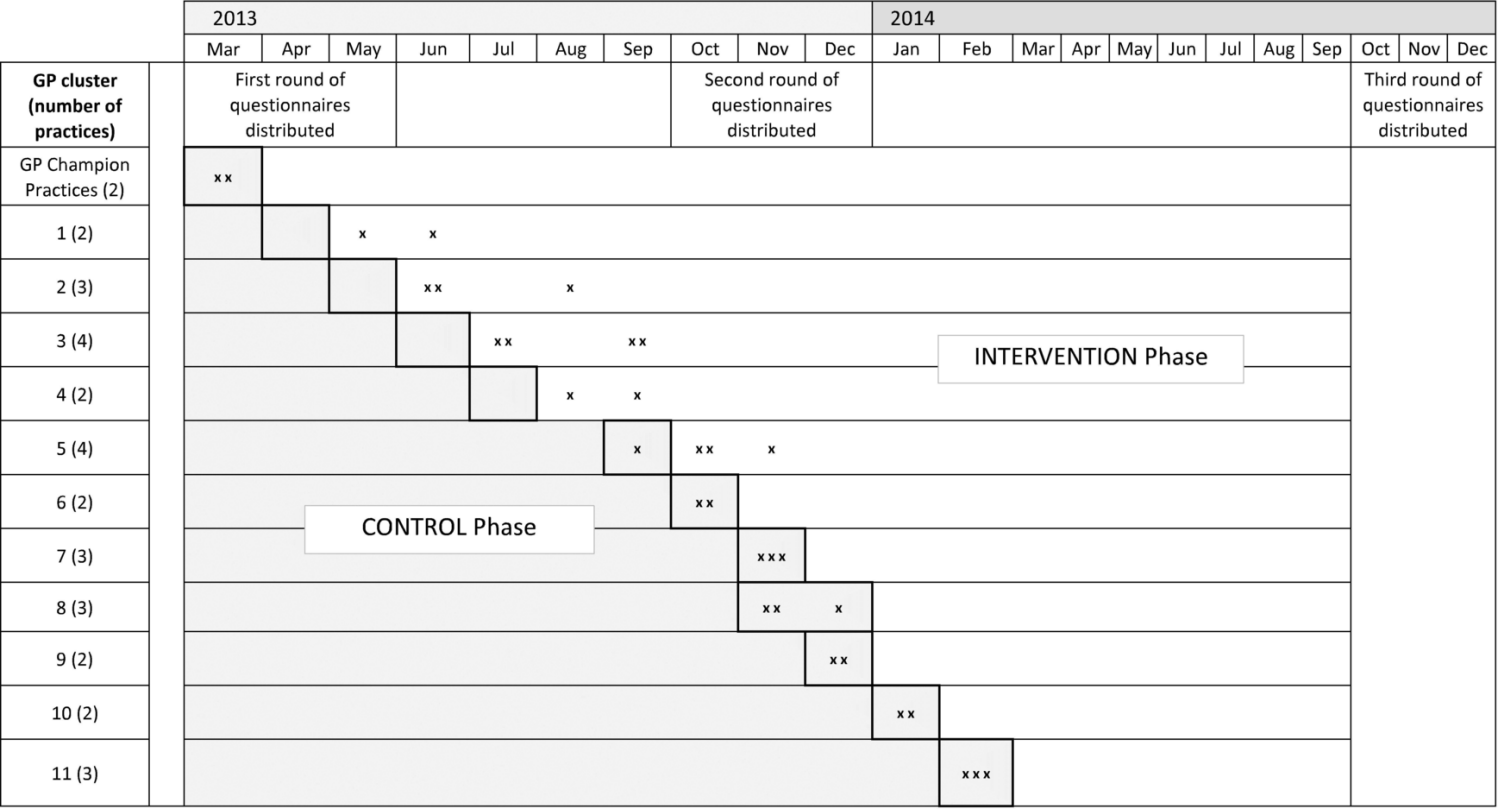
Stepped wedge study design with dates of practice training as planned and undertaken (x).

We undertook a randomised stepped wedge trial, a form of cluster randomised trial.^18 19^ This design gives all participating general practices the opportunity to use the intervention during the study, and careful analysis separates the effects of the intervention from trends, both seasonal and longitudinal.^20 21^ We invited all 77 general practices within Abertawe Bro Morgannwg University Health Board to participate, recruited the 32 (42%) practices who volunteered, and grouped them into 11 clusters based on existing community networks. Swansea Trials Unit used random numbers to set the order in which practice clusters received the intervention. We concealed that allocation from practices until shortly before implementation. All participating practices began as control practices without the trial intervention. As the trial progressed, the number of intervention practices increased each month and the number of control practices fell (figure 1). Though study practices received QOF payments throughout the study as an incentive to participate, they did not have to identify or review high-risk patients until the PRISM software was installed. Thereafter, they used PRISM to identify patients who were at high risk of unscheduled admission. As we used routinely available anonymised data for our primary outcome, we did not need to consent patients formally to participate in the trial. Our single designated enrolment date is therefore the first day on which the PRISM tool was made available to support care delivery by general practices (30 May 2013).

We also undertook qualitative research to explore prior beliefs, implementation and patterns of use, reported in detail elsewhere.^18 19 22^

### Intervention

The PRISM intervention (table 1) allows health professionals in general practice to view individual patients’ scores summarising their risk of emergency admission during the following year via a secure website. The NHS Wales Informatics Service (NWIS) used the anonymised data of 300 000 people in Wales (10% of the population) to develop the PRISM risk model and test it for accuracy of prediction.^23^ The routine data available for testing, included inpatient, outpatient and general practice data, alongside a deprivation index—the Welsh Index of Multiple Deprivation (WIMD). The final model included the 37 variables with the highest predictive power. NWIS update scores monthly and stratify them within each practice thus: risk group 1 comprises the 80% of the practice population with the lowest scores; risk group 2 the 15% with the next high scores; risk group 3 the 4.5% with the next high scores and risk group 4 the 0·5% at highest risk of emergency hospital admission. The intervention also included a user friendly handbook, 2 hours of practice-based training, clinical support through two locally appointed ‘general practitioner (GP) champions’ and a ‘help desk’ accessible by telephone or email. We did not specify how or when practices should use the software to manage care. The training recommended that practices regularly review those at medium to high risk and select patients for active management to prevent worsening health and emergency admission. Suggestions for proactive care discussed in training included home visits, practice-initiated appointments, review in multidisciplinary team meetings and coordinating care with community and secondary care services.

**Table 1:** Components of the intervention

Web-based PRISM tool	Installed on computers in each practice and activated when the practice began the intervention.
Practice-based training	Two-hour session delivered in the practice by a GP champion to the nominated lead GP for PRISM, the practice manager and any other interested staff.
GP champions	Two local GPs employed for two sessions per month to support practices in clinical use of PRISM.
Technical help desk	Telephone and email support provided in office hours by NHS Wales Informatics Service (NWIS) to answer enquiries about technical aspects of using PRISM.
PRISM handbook	User friendly 25-page handbook explaining how to set up and access PRISM, demonstrating the range of functions available in PRISM and recommending how to use it within the practice.
Targeted care	Practices were free to plan tailored care for patients at high risk. QOF payments required identification and active management of patients at high risk.

GP, general practitioner; PRISM, predictive risk stratification model; QOF, Quality and Outcomes Framework.

### Outcomes

We compared between intervention and control phases:

#### Primary outcome

Emergency hospital admissions

#### Secondary outcomes

- Attendances at hospital emergency departments (EDs).
- Outpatient attendances
- Primary care events
- Length of stay following emergency admission.
- Health-related quality of life assessed by the SF-12^24^.
- Patient satisfaction assessed by the quality of care monitor (QCM)^25^.
- Costs of implementing PRISM, both direct and through changes in healthcare.

We also compared deaths between phases to check for unexpected effects.

### Data collection

We accessed routine data on healthcare use for all patients registered with participating practices at the start of the study period. We deemed patients leaving practices for any reason to have left the study and did not replace them by those registering during the study. We sent postal questionnaires to a stratified random sample of study patients.

### Anonymised linked data

We used routine data from the Secure Anonymised Information Linkage (SAIL) databank^26^ to compare between intervention and control phases—emergency, secondary and primary care used by patients across the spectrum of risk. SAIL includes routine data on deaths (Office of National Statistics), emergency admissions (Emergency Department Data Set), secondary care (Patient Episode Database for Wales) and general practice data. We displayed posters in each participating general practice offering patients the opportunity to withdraw from this analysis and removed all patients who so dissented. We linked PRISM scores from general practice to health service use data for all patients who had not dissented.

### Postal questionnaires

We sent postal questionnaires to a random sample of patients, stratified by risk level, at three time points during the study—before, during and after implementation (figure 1). We weighted the sample to include proportionally more patients from risk groups 3 and 4—the groups at highest risk, but also the smallest. The questionnaire comprised the SF-12^24^ to measure health-related quality of life and the QCM^25^ to measure patient satisfaction. At each time point, we provided 2240 questionnaires for practices to distribute to patients, aiming for 800 responses. Practices screened out patients recently deceased, moved or otherwise unsuitable, resulting in 5232 questionnaires posted to patients.

### Data on use of PRISM

We monitored the use of PRISM (frequency, duration and purpose) through practice staff questionnaires and interviews at 9 and 18 months, and data on PRISM logins supplied by NWIS.

### Statistical methods

We undertook analysis by treatment allocated, and assigned participants’ events to control or intervention phases for analysis according to the planned implementation date of the PRISM tool in their general practice. We estimated intracluster correlation coefficients (ICC) between participants in the same study practice. Using Stata V.14, we analysed outcomes, expressed as rates based on counts but taking account of phase duration, by linear mixed models which always included the effect of PRISM. We considered, and where necessary adjusted for, the following covariates: gender, age in years, WIMD score and its separate health component (both from 2011), initial PRISM score, season and trend. We treated study practice as a random factor, and defined a second independent random factor to account for paired ‘control’ and ‘intervention’ observations from participants. To mitigate pronounced skewness in event-based data, we analysed and present log-transformed data. Modelling progressed by eliminating all covariates found to be not statistically significant, starting with the least significant and concluded when all remaining covariates were statistically significant. We examined the assumption of normality by residual diagnostics. We analysed SF12 and QCM scores by similar repeated-measures linear models.

### Economic methods

We undertook economic evaluation from the perspective of the UK NHS. We estimated the costs of PRISM implementation (including setup, training, GP staff time, IT support and maintenance), and primary and secondary care use (including emergency admissions, ED attendances, outpatient visits and all inpatient stays) in Pounds Sterling from published unit costs for 2015,^27^ GP staff interviews and questionnaires and SAIL. We estimated the budgetary effect on the NHS of adopting PRISM scoring in primary care based on the total cost per 100 000 patients registered in participating practices over the trial period.

### Patient involvement

We recruited two patient representatives from a local service user group ‘Service Users with Chronic Conditions Encouraging Sensible Solutions’ (SUCCESS).^28^ They were members of the research management team throughout the trial and maintained links with the wider SUCCESS group for input and support. We worked closely with them in designing the trial, the intervention and patient materials including the information sheet, consent form and research instruments. Both representatives are coauthors.

## Results

### Participant flow

We have a history of NHS contacts for 230 114 participants from 1 February 2013 until 30 September 2014. This includes the period of at least 4 months before PRISM implementation and up to 16 months after. Fifteen people spent the whole study period in hospital. We therefore included outcomes from routine NHS data on 230 099 participants, of whom 8034 left their practices during their control phase and 15 676 during their intervention phase. We did not include these patients after they left, irrespective of where they reregistered.

### Baseline characteristics

Table 2 summarises baseline characteristics of the study population by risk groups. Mean age increases from 37 years in the lowest risk group to 70 in the highest; higher risk groups include more women and WIMD scores slightly increase with risk across groups. Initial PRISM scores were available on almost all patients, with considerable overlap between risk groups, due to variation between practices in thresholds.

**Table 2:** Baseline characteristics

PRISM risk group	n=	Proportion female	Age in years mean (SD)	WIMD scores Mean (SD)		PRISM score	
				Overall	Health	mean (SD)	min; max
AlI*	230 099	0.501	41.2 (23.4)	24.0 (16.3)	27.1 (22.1)	6.53 (6.89)	1.68; 99.84
Risk group 1	182 955	0.490	36.6 (20.5)	23.3 (16.0)	26.3 (21.8)	4.16 (1.61)	1.68; 11.58
Risk group 2	34 311	0.540	56.7 (25.2)	26.7 (17.2)	30.0 (23.0)	11.49 (3.18)	4.07; 23.11
Risk group 3	10 292	0.565	69.8 (21.4)	27.3 (17.2)	31.0 (22.9)	26.15 (7.97)	6.76; 60.48
Risk group 4	1129	0.530	70.2 (22.1)	27.6 (17.1)	31.6 (22.5)	60.87 (13.63)	19.40; 99.84

*n=1412 (0·6%) people did not have a PRISM Score recorded at baseline and were therefore not assigned to a risk group.

PRISM, predictive risk strtification model; WIMD, Welsh Index of Multiple Deprivation.

### Use of PRISM

NWIS reported that 58 practice staff (an average of 1.8 per practice) registered to use PRISM in the intervention phase and logged in on 260 occasions (an average of 8.1 per practice). The average time spent using the PRISM website itself was 60 min (from user questionnaires). However, interviews revealed that most practices printed lists of patients from PRISM, chiefly to use for QOF-related activities.

### Effects

Adjusting log-transformed data for length of time in each phase and all other significant covariates led to greater changes in risk groups 3 and 4 than in risk groups 1 and 2, owing in part to the small sizes of these groups. Thus, we found an increase in our primary outcome of emergency admissions per participant per year at risk of 1% in the intervention phase (table 3). Emergency admissions were higher in the intervention phase, and the effect increased with predicted risk.

**Table 3:** Clinical and cost outcomes for all participants by phase*

Clinical/cost outcome	Intervention phase	Control phase	Adjusted comparison†‡§			
	Mean (SD)	Mean (SD)	Original	Transformed		
			Δ	Δ_L_	(95% CI)	P values
Emergency hospital admissions: all	0.17 (2.08)	0.16 (2.16)	0.005	0.011	(0.010 to 0.013)	<0.001
Risk group 1	0.07 (0.75)	0.06 (0.70)	0.002	0.006	(0.005 to 0.007)	<0.001
Risk group 2	0.37 (3.70)	0.29 (2.36)	0.053	0.026	(0.021 to 0.031)	<0.001
Risk group 3	1.05 (5.44)	1.07 (6.90)	-0.035	0.061	(0.045 to 0.076)	<0.001
Risk group 4	3.30 (10.92)	3.48 (15.40)	-0.063	0.110	(0.040 to 0.179)	0.002
ED attendances: all	0.36 (1.79)	0.36 (2.10)	0.006	0.030	(0.028 to 0.032)	<0.001
Risk group 1	0.27 (1.09)	0.27 (1.10)	0.005	0.026	(0.024 to 0.029)	<0.001
Risk group 2	0.54 (2.75)	0.49 (1.90)	0.044	0.040	(0.034 to 0.047)	<0.001
Risk group 3	1.17 (4.19)	1.24 (6.24)	-0.066	0.058	(0.042 to 0.075)	<0.001
Risk group 4	3.04 (8.40)	3.24 (14.44)	-0.134	0.074	(0.005 to 0.143)	0.035
GP event days: all	14.08 (32.59)	14.10 (23.12)	0.139	0.011	(0.007 to 0.014)	<0.001
Risk group 1	9.42 (13.67)	9.32 (12.55)	0.044	0.023	(0.018 to 0.027)	<0.001
Risk group 2	29.24 (43.28)	27.78 (24.96)	0.301	0.042	(0.035 to 0.049)	<0.001
Risk group 3	47.19 (109.4)	46.59 (53.81)	1.973	-0.031	(−0.044 to −0.018)	<0.001
Risk group 4	67.30 (68.66)	78.72 (120.98)	−8.383	-0.090	(−0.139 to −0.041)	<0.001
Outpatients visits: all	1.72 (9.75)	1.70 (9.98)	0.035	0.055	(0.051 to 0.058)	<0.001
Risk group 1	1.09 (5.63)	1.02 (4.54)	0.058	0.056	(0.053 to 0.060)	<0.001
Risk group 2	3.42 (11.20)	3.46 (13.72)	−0.015	0.050	(0.040 to 0.060)	<0.001
Risk group 3	6.55 (27.09)	6.69 (27.85)	−0.006	0.025	(0.005 to 0.046)	0.016
Risk group 4	13.50 (65.56)	13.83 (59.87)	−0.147	-0.007	(−0.076 to 0.063)	0.851
Days in hospital: all	0.73 (5.89)	0.79 (9.93)	−0.010	0.029	(0.026 to 0.031)	<0.001
Risk group 1	0.26 (3.32)	0.28 (7.58)	0.006	0.015	(0.013 to 0.017)	<0.001
Risk group 2	1.62 (8.71)	1.63 (12.16)	0.002	0.066	(0.056 to 0.076)	<0.001
Risk group 3	5.16 (15.31)	5.61 (22.57)	−0.148	0.150	(0.120 to 0.180)	<0.001
Risk group 4	13.38 (25.91)	15.15 (33.93)	−1.121	0.197	(0.073 to 0.320)	<0.001
Total health care cost per patient per year in £: all	1548 (6226)	1535 (7260)	76		(46 to 106)	<0.001
Risk group 1	868 (3896)	809 (4785)	61		(38 to 84)	<0.001
Risk group 2	3267 (9119)	3183 (9687)	137		(22 to 252)	0.020
Risk group 3	7027 (14 538)	7429 (17 412)	134		(−210 to 477)	0.446
Risk group 4	15570 (23 733)	1587 9(26 476)	908		(−810 to 2625)	0.300

*Numbers analysed (intervention/control). All risk groups: 220683/230087; risk group 1: 176214/182952; risk group 2: 32929/34307; risk group 3: 9352/10288; risk group 4: 897/1128.

†The variables are summarised and analysed using event rates per year at risk; online supplementary table 1 shows significant covariates and factors.

‡Covariates considered were: phase; gender; age; PRISM score; WIMD score; WIMD health component on or near 1 February 2013 and seasonality and trend scores for phases.

§The comparison between phases is summarised as additive phase effect in the dependent variable estimated from mixed linear models: Δ in original units and Δ _L_ in log-transformed data with ln(1+*y*) in place of *y*.

ED, emergency department; GP, general practitioner; PRISM, predictive risk stratification model; WIMD, Welsh Index of Multiple Deprivation.

10.1136/bmjqs-2018-007976.supp1 Supplementary data

The number of ED attendances per participant per year at risk was 3% higher in the intervention phase, an effect that was consistent across risk groups and increased with predicted risk. Outpatient attendances increased by 5% per participant per year in the intervention phase, owing mainly to an increase in the two lowest risk groups. We found an increase of 1% in the number of days on which GPs recorded activity per participant per year in the intervention phase, although this effect was not consistent across risk groups. Bed days increased by 3% per participant per year in the intervention phase; this effect was consistent across risk groups and increased with predicted risk.

We analysed data from 2362 self-report questionnaires from 1403 distinct patients: achieving a 45.1% response rate. Table 4 shows no difference in SF12 mental health component quality of life scores, but improved physical health component scores in respondents in the intervention phase, with a trend towards greater improvement in those in the higher risk groups. Satisfaction scores were slightly lower in the intervention phase with no clear pattern across risk groups.

**Table 4:** Survey outcomes for sampled participants by phase

Survey outcome	Intervention phase		Control phase		Adjusted comparison*†‡		
	Mean (SD)	(n)	Mean (SD)	(n)	Δ	(95%CI)	P values
SF12 mental health component: all	43.77 (9.67)	(1410)	44.62 (9.47)	(662)	−0.720	(−1.469 to 0.030)	0.060
Risk group 1	49.44 (8.34)	(242)	49.03 (8.07)	(83)	1.736	(0.106 to 3.366)	0.037
Risk group 2	43.72 (8.97)	(381)	45.18 (8.85)	(163)	−0.802	(−2.222 to 0.618)	0.268
Risk group 3	42.54 (9.53)	(659)	44.13 (9.80)	(322)	−1.187	(−2.317 to -0.056)	0.040
Risk group 4	39.61 (10.35)	(127)	41.45 (9.10)	(94)	−1.911	(−4.079 to 0.258)	0.084
SF12 physical health component: all	41.72 (9.20)		40.07 (7.30)		1.465	(0.774 to 2.157)	<0.001
Risk group 1	34.41 (9.09)		36.77 (7.62)		−4.385	(−6.106 to -2.664)	<0.001
Risk group 2	41.90 (8.18)		40.65 (7.12)		0.882	(−0.345 to 2.108)	0.159
Risk Group 3	43.54 (8.45)		40.18 (7.15)		3.205	(2.218 to 4.191)	<0.001
Risk group 4	45.56 (8.76)		41.61 (7.11)		4.103	(2.230 to 5.977)	0.035
SF6D score: all	0.638 (0.068)	(1467)	0.638 (0.072)	(692)	−0.0002	(−0.008 to 0.004)	0.584
Risk group 1	0.629 (0.069)	(247)	0.642 (0.077)	(83)	-0.019	(−0.034 to -0.003)	<0.001
Risk group 2	0.639 (0.072)	(399)	0.641 (0.075)	(166)	−0.003	(−0.015 to 0.010)	0.649
Risk group 3	0.641 (0.067)	(690)	0.638 (0.070)	(341)	0.002	(−0.006 to 0.010)	0.628
Risk group 4	0.635 (0.060)	(130)	0.633 (0.069)	(102)	0.002	(−0.013 to 0.017)	0.787
QCM score: all	4.20 (0.74)	(1408)	4.27 (0.70)	(660)	−0.074	(−0.133 to -0.015)	0.014
Risk group 1	4.19 (0.72)	(189)	3.96 (0.86)	(62)	0.169	(−0.030 to 0.368)	0.095
Risk group 2	4.24 (0.69)	(391)	4.28 (0.72)	(162)	−0.080	(−0.191 to 0.031)	0.156
Risk group 3	4.20 (0.74)	(697)	4.36 (0.61)	(340)	−0.107	(−0.186 to -0.029)	0.008
Risk group 4	4.04 (0.87)	(130)	4.13 (0.76)	(96)	−0.100	(−0.286 to 0.086)	0.291

*online supplementary table 2 shows significant covariates and factors.

†Covariates considered were: phase; gender; age; PRISM score; WIMD score; WIMD health component on or near 1 February 2013) and seasonality and trend scores for phases.

‡The comparison between phases is summarised as an additive phase effect Δ in the same units as the dependent variable, estimated from mixed linear models.

PRISM, predictive risk stratification model; QCM, quality of care monitor; WIMD, Welsh Index of Multiple Deprivation.

### Harms

We found no evidence of any difference in mortality rates between phases.^19^

### Technical performance

A systematic review in 2014 (during this trial) of emergency admission risk prediction models identified 27 tools, of which 18 predicted emergency hospital admission within 12 months^29^. The models that predicted best, as measured by the area under the receiver operating characteristic curve (the ‘c statistic’), included routine measures of previous healthcare use, multimorbidity and prescribing, as PRISM did. Using data from 51 600 patients with both an early PRISM score and a sufficient control phase, we found that PRISM achieved good technical performance with c=0.749, comparable to the previous risk prediction tools. However, it generally under-predicted risk at higher risk levels and over-predicted risk at the lowest risk level.

### Health economics

#### Cost of implementing PRISM

We estimated that implementing PRISM cost £25 350 (£792 per practice) in the first year: £1423 for activation; £9709 for training; £952 for annual running and maintenance costs; £1024 for PRISM software updates and £12 242 for practice staff using PRISM. We inferred that, without activation and initial training, (undiscounted) PRISM operating costs for subsequent years were £14 218 (£444 per practice). As we studied 230 099 participants, these costs equate to an initial £0·11 per participant and £0·06 per participant per year thereafter.

#### Primary and secondary healthcare costs

Comparison of control and intervention phases showed an adjusted increase of £76 (95% CI £46 to £106; p<0·001) in healthcare costs over 1 year in the intervention phase. These differences were statistically significant across the population and within risk groups 1 and 2, though not risk groups 3 and 4. Similar statistically significant differences were apparent in log-transformed costs. We reported health economic results in detail elsewhere.^19^

#### Cost-effectiveness analysis

Though PRISM itself cost only £0·11 per participant in the first year, it was significantly less effective and significantly more costly to the NHS than usual care. In economic terms, the control phase dominates, so incremental cost-effectiveness ratios (ICERs) are not valid.

#### Cost-utility analysis

Estimated SF-6D utilities were slightly lower for the intervention phase (mean 0·6380) than the control phase (mean 0·6382). After adjusting for covariates, the difference was not statistically significant (p=0·584). Thus, the control treatment again dominates, and ICERs are not valid. We used a cost-effectiveness acceptability curve and a willingness-to-pay threshold of £20 000 to estimate the probability of PRISM being cost effective as only 46%.

#### Budget impact of PRISM during the study

The rise in healthcare costs that followed the implementation of PRISM across the trial area increased the estimated budget by £7·59 million per 100 000 population per annum (95% CI: £4·58 million to £10·60 million).

## Discussion

### Principal findings

We found that introducing PRISM software alongside a national policy initiative to prioritise the care of people at highest risk of emergency admission to hospital increased emergency admissions to hospitals, time spent in hospital and the use of other primary and secondary care, especially those with the highest risk scores. There was evidence of improved physical health-related quality of life, but satisfaction scores were lower, and there was no net gain in patient utility. Though the direct cost of the intervention was small, it increased the use and cost of NHS services. Hence, PRISM was inferior to routine practice. We estimate that PRISM increased total healthcare costs by £7·6 million per 100 000 population per annum.

### Strengths and limitations

Our stepped wedge trial design allocated clusters of GP practices to receive PRISM software at random points over a year. Our evaluation linked routine data over 2 years. Together, these novel and relatively inexpensive methods enabled us to conduct a powerful and rigorous evaluation of this population-level intervention in nearly a quarter of a million people. We also linked self-completed questionnaires for a large sample of patients anonymously to their routine data outcomes, thus evaluating quality of life and satisfaction as well as health service use. We recognise the dynamic context within which we conducted this study, with political and clinical changes before, during and after recruitment. Fortunately, our trial design is well suited to such circumstances, often encountered by evaluative studies in healthcare.^30^ This design compared populations in parallel and over time, thus adjusting for background trends in service use and population ageing. Unfortunately, though there were plans to give practice staff greater access to multidisciplinary community resource teams, this did not happen.

### Implications for policy and practice

This research addresses a major policy concern for the NHS—how best to manage patients in the community to avoid unnecessary, disruptive and costly emergency admissions. The use of tools to predict the risk of emergency admission is widely advocated as a core component of models to care for long-term conditions. However, there is debate about whether to focus on those at the highest level of risk, as these are few in number.^31 32^ Despite these concerns, targeting those at the highest levels of risk has become routine practice—in Wales through QOF measures and in England through the enhanced service. These policy initiatives assume that identifying patients at high risk of emergency admission will facilitate interventions to improve their health and reduce unplanned episodes of care.^31^ Unfortunately, our trial showed the opposite effect, in common with analogous but weaker studies. Stokes *et al*’s systematic review of 36 case management initiatives for patients at risk of hospitalisation, but not necessarily identified through an emergency admission risk prediction tool, found no positive effects on service use, but a small improvement in patient satisfaction.^17^ A controlled before-and-after study of multidisciplinary case management for high-risk patients identified by risk tools, meeting the requirements of the English Unscheduled Admissions Enhanced Service, also showed no evidence of patient benefit.^32^ A study of virtual wards—a multidisciplinary team offering ‘hospital at home’ to patients at high risk of admission—also did not reduce emergency admissions.^33^

Thus our study strengthens the evidence that current approaches using emergency admission risk prediction tools to identify and support patients do not reduce health service use and costs. Policy makers should therefore consider alternative approaches. For example, Wallace *et al* concluded that alternatives to case management should focus on reducing the length of hospital inpatient stay while preventing readmission.^34^

We hypothesise that the combination of PRISM and QOF incentives to focus on those at high risk of emergency admission to hospital alerted GPs and practice staff to unmet needs and lowered their threshold for admitting patients to hospital. Predictive risk stratification is a tool which needs effective interventions to avoid emergency episodes of care. Such interventions need explicit models of how they will work and for whom, and rigorous evaluation of their clinical and cost effectiveness, before implementation.

## Conclusions

Introduction of predictive risk stratification increased emergency hospital admissions, use of other NHS services and therefore costs, at each risk level within a large general practice population without evidence of benefits to patients or the NHS.
