# Supplementary material for: Effects and costs of implementing predictive risk stratification in primary care: a randomised stepped wedge trial
Source: BMJ Qual Saf. 2018 Nov 5;28(9):697–705. doi: 10.1136/bmjqs-2018-007976 (PMC6820297; doi:10.1136/bmjqs-2018-007976)
Supplement: Supplementary data [file bmjqs-2018-007976supp001.pdf]

BMJQS-2018-007976

Effects and costs of implementing predictive risk stratification in primary care: a randomised stepped wedge trial.

Supplementary Tables

Supplementary Table 1: Significant covariates and factors for analyses in Table 2.

**Emergency Hospital Admissions**								
All	Δ	Age	Gender (p=0.033)	PRISM	Seasonality	Trend (p=0.012)	WIMD	
	Δ_L	PRISM	Seasonality	Trend	WIMD			
Risk Group 1	Δ	Age	PRISM	Seasonality				
	Δ_L	Age	PRISM	Seasonality	Trend	WIMD HC (p=0.007)		
Risk Group 2	Δ	Age	PRISM	Seasonality	Trend (p=0.012)	WIMD (p=0.001)	WIMD HC (p=0.028)	
	Δ_L	Age (p=0.001)	PRISM	Seasonality	Trend (p=0.039)	WIMD	WIMD HC (p=0.044)	
Risk Group 3	Δ	Age	Gender (p=0.017)	PRISM	Seasonality	WIMD HC (p=0.002)		
	Δ_L	Age	Gender	PRISM	Seasonality	WIMD		
Risk Group 4	Δ	PRISM (p=0.003)	Seasonality					
	Δ_L	PRISM	Seasonality (p=0.003)					
**Emergency Department Attendances**								
All	Δ	Age	Gender	PRISM	Seasonality	Trend		

Snooks H, *et al. BMJ Qual Saf* 2019; 0:697–705. doi: 10.1136/bmjqs-2018-007976

	Δ_L	Age	Gender	PRISM	Trend	WIMD		
Risk Group 1	Δ	Age	Gender	PRISM	Seasonality	Trend	WIMD	
	Δ_L	Age	Gender	PRISM	Seasonality	Trend	WIMD	
Risk Group 2	Δ	Age	PRISM	Seasonality				
	Δ_L	Age	Gender (p=0.009)	PRISM	Seasonality	WIMD (p=0.029)		
Risk Group 3	Δ	PRISM	Seasonality					
	Δ_L	Age (p=0.003)	PRISM	Seasonality				
Risk Group 4	Δ	PRISM	Trend					
	Δ_L	PRISM						
**GP Event Days**								
All	Δ	Age	Gender	PRISM	Seasonality	Trend	WIMD	WIMD HC
	Δ_L	Age	Gender	PRISM	Seasonality	Trend	WIMD	WIMD HC
Risk Group 1	Δ	Age	Gender	PRISM	Seasonality	Trend	WIMD	WIMD HC
	Δ_L	Age	Gender	PRISM	Seasonality	Trend	WIMD	WIMD HC
Risk Group 2	Δ	Age	Gender	PRISM	Seasonality	Trend	WIMD	WIMD HC
	Δ_L	Age	Gender	PRISM	Seasonality	Trend	WIMD	WIMD HC
Risk Group 3	Δ	Age	PRISM	Seasonality	WIMD (p=0.011)			
	Δ_L	Age	PRISM	Seasonality	Trend	WIMD	WIMD HC	
Risk Group 4	Δ	Age (p=0.012)	PRISM (p=0.019)					
	Δ_L	Age (p=0.003)	Seasonality	WIMD	WIMD HC (p=0.001)			
**Outpatients Visits**								
All	Δ	Age	Gender	PRISM	Seasonality	Trend	WIMD	
	Δ_L	Age	Gender	PRISM	Seasonality	WIMD		
Risk Group 1	Δ	Age	Gender	PRISM	Seasonality	Trend (p=0.001)	WIMD	
	Δ_L	Age	Gender	PRISM	Seasonality	WIMD		
Risk Group 2	Δ	Age	Gender (p=0.019)	PRISM	Seasonality	Trend (p=0.001)	WIMD	
	Δ_L	Age	Gender	PRISM	Seasonality	WIMD		
Risk Group 3	Δ	PRISM	Seasonality					
	Δ_L	Age	Gender	PRISM	WIMD			

Snooks H, *et al. BMJ Qual Saf* 2019; 0:697–705. doi: 10.1136/bmjqs-2018-007976

		(p=0.001)						
Risk Group 4	Δ	Seasonality (p=0.025)	Trend (p=0.007)					
	Δ_L	Age	PRISM (p=0.024)	Seasonality (p=0.028)	Trend (p=0.045)	WIMD (p=0.020)		
**Days In Hospital**								
All	Δ	Age	Gender (p=0.006)	PRISM	Seasonality	WIMD		
	Δ_L	Age	Gender (p=0.014)	PRISM	Seasonality	Trend	WIMD	
Risk Group 1	Δ	Age	PRISM	Seasonality				
	Δ_L	Age	PRISM	Seasonality (p=0.002)	Trend			
Risk Group 2	Δ	Age	PRISM	Seasonality	WIMD			
	Δ_L	Age	PRISM	Seasonality	Trend (p=0.026)	WIMD		
Risk Group 3	Δ	Age	Gender (p=0.017)	PRISM	Seasonality	WIMD HC		
	Δ_L	Age	Gender (p=0.003)	Seasonality	WIMD HC			
Risk Group 4	Δ	Age (p=0.022)	PRISM					
	Δ_L	Age (p=0.008)	PRISM					

p<0.001 unless otherwise stated.

PRISM: Prism Score at or near 1.2.2013
WIMD Welsh Index of Multiple Deprivation
WIMD HC Welsh Index of Multiple Deprivation – Health Component

Snooks H, *et al. BMJ Qual Saf* 2019; 0:697–705. doi: 10.1136/bmjqs-2018-007976

Supplementary Table 2: Significant covariates and factors for analyses in Tables 3 & 5.

**SF12 Mental Health Component**					
All	Age (p=0.003)	PRISM	WIMD HC		
Risk Group 1	Age (p=0.029)	PRISM (p=0.016)	WIMD		
Risk Group 2	Age	WIMD (p=0.031)			
Risk Group 3	Age (p=0.003)	Gender (p=0.048)	PRISM (p=0.027)	WIMD HC (p=0.001)	
Risk Group 4	Age (p=0.007)	WIMD HC (p=0.013)			
**SF12 Physical Health Component**					
All	PRISM	WIMD			
Risk Group 1	PRISM	WIMD			
Risk Group 2	Age (p=0.001)				
Risk Group 3	Age	PRISM	WIMD		
Risk Group 4	WIMD HC (p=0.043)				
**SF6D**					
All	Age	PRISM (p=0.028)	WIMD HC (p=0.027)		
Risk Group 1	Age				
Risk Group 2	Age				
Risk Group 3	Age (p=0.003)				
Risk Group 4	Age	WIMD HC (p=0.049)			
**Quality of Care Monitor Score**					
All	Age	PRISM (p=0.007)	WIMD (p=0.018)	WIMD HC (p=0.007)	
Risk Group 1	Age(p=0.009)				
Risk Group 2	Age (p=0.005)	WIMD (p=0.026)	WIMD HC (p=0.009)		
Risk Group 3	Age (p=0.019)				
Risk Group 4	*None*				
**Healthcare Costs**					
All	Age	PRISM	Seasonality	Trend (p=0.007)	WIMD
Risk Group 1	Age	Gender (p=0.002)	PRISM	Seasonality	WIMD
Risk Group 2	Age	PRISM	Seasonality	Trend (p=0.026)	WIMD
Risk Group 3	Age	Gender	PRISM	Seasonality	WIMD HC
Risk Group 4	PRISM	WIMD (p=0.010)	WIMD HC (p=0.038)		

Snooks H, *et al. BMJ Qual Saf* 2019; 0:697–705. doi: 10.1136/bmjqs-2018-007976

$p<0.001$ unless otherwise stated.

PRISM:	Prism Score at or near 1.2.2013
WIMD	Welsh Index of Multiple Deprivation
WIMD HC	Welsh Index of Multiple Deprivation – Health Component

Snooks H, *et al. BMJ Qual Saf* 2019; 0:697–705. doi: 10.1136/bmjqs-2018-007976
